# Sequencing and Analysis of the Complete Mitochondrial Genome of *Lentipes ikeae*

**DOI:** 10.3390/ani14060943

**Published:** 2024-03-19

**Authors:** Cheng-He Sun, Yang-Liang Gu, Da-Wei Liu, Hong-Wei Du, Chang-Hu Lu

**Affiliations:** 1 College of Life Sciences, Nanjing Forestry University, Nanjing 210037, China; luchanghu@njfu.com.cn; 2 National Key Laboratory of Water Environmental Simulation and Pollution Control, South China Institute of Environmental Sciences, Ministry of Ecology and Environment, Guangzhou 510530, China; guyangliang@scies.org (Y.-L.G.); duhongwei@scies.org (H.-W.D.); 3 Faculty of Criminal Science and Technology, Nanjing Police University, Nanjing 210023, China; dwliu@nfpc.edu.cn

**Keywords:** Gobiidae, Sicidiinae, mitochondrial genome, phylogenetic relationships

## Abstract

**Simple Summary:**

In brief, the mitochondrial genome of *L. ikeae* has a total length of 16,498 bp and encodes 13 PCGs, 22 transfer RNA genes, two ribosomal RNA genes, and a D-loop (control) region. Gene rearrangement is not observed. The mitochondrial genome of *L. ikeae* exhibits an AT preference, with AT skew > 0 and GC skew < 0 across the entire genome. The phylogenetic relationships of Sicydiinae based on 13 mitochondrial PCG sequences are *Sicydium* + (*Stiphodon* + (*Sicyopus* + *Lentipes*)) + *Sicyopterus*, indicating that *Sicydium*, *Sicyopterus*, *Lentipes*, and *Stiphodon* are all monophyletic groups.

**Abstract:**

We sequenced and analyzed the complete mitochondrial genome of *Lentipes ikeae* and explored the phylogenetic relationships among Sicydiinae based on mitochondrial genome sequences. The complete mitochondrial genome sequence of *L. ikeae* was determined using the Illumina HiSeq X Ten sequencing platform, and the gene structural characteristics and base composition were analyzed. Based on the mitochondrial genome sequences of 28 Sicydiinae species published in GenBank and mitochondrial protein-coding genes (PCGs), *Acanthogobius flavimanus* (Gobionellinae) was selected as an outgroup to construct phylogenetic trees of Sicydiinae using the maximum likelihood and Bayesian inference methods. The mitochondrial genome of *L. ikeae* (GenBank number: OP764680) has a total length of 16,498 bp and encodes 13 PCGs, 22 transfer RNA genes, two ribosomal RNA genes, and a D-loop (control) region. Gene rearrangement is not observed. The mitochondrial genome of *L. ikeae* exhibits an AT preference, with AT skew > 0 and GC skew < 0 across the entire genome. The phylogenetic relationships of Sicydiinae based on 13 mitochondrial PCG sequences are *Sicydium* + (*Stiphodon* + (*Sicyopus* + *Lentipes*)) + *Sicyopterus*, indicating that *Sicydium*, *Sicyopterus*, *Lentipes*, and *Stiphodon* are all monophyletic groups.

## 1. Introduction

The complexity of fishery ecosystems requires an accurate understanding of species. However, due to their different growth environments and developmental stages, aquatic organisms often exhibit different morphological characteristics, such as “the same species but different forms” and “different species and the same forms”, as well as commonly existing hidden taxonomic units, which complicates traditional morphological research. The morphology of gobies is diverse and widely distributed [1,2]. Their morphology rapidly differentiates and forms within an extremely short period of time, which is also common in other groups. It is difficult to reconstruct the phylogenetic relationships of gobies using general molecular markers. Therefore, it is extremely important to clarify the evolutionary relationships between them.

*Lentipes ikeae* Keith, Hubert, Busson & Hadiaty, 2014 belonging to the order Gobiiformes, family Gobiidae, subfamily Sicydiinae [1]. They are mainly distributed in Cisolok, Kab Sukabumi, Java, and Indonesia [1,3] and occur in fast-flowing mountain streams with high gradients and small yet clear, oxygen-rich streams with rocky bottoms, typically at an altitude of 310–488 m. *L. ikeae* is an omnivorous species that accepts frozen red worms and mainly eats shrimp and water fleas, and even algae when there are no other food sources. In its combat mode, it has bright colors and important ornamental value, and it is a water quality indicator species [2,4].

The mitochondrial genome can autonomously replicate under the control of nuclear genes, and it is characterized by maternal inheritance, a small size, and a fast evolution rate. Further, it can be easily PCR-amplified. With the continuous development of sequencing technology, mitochondrial genomes have been widely used to study the phylogeny of various fish species and serve as important markers for classification and molecular evolution [5,6,7]. In recent years, many researchers have used mitochondrial genome data to analyze the phylogeny of Sicydiinae [8,9,10]. The mitochondrial genome sequences of 28 species of Sicydiinae have been published in the GenBank database, and the most complete sequencing data cover five genera of Sicydiinae.

In this study, we sequenced and analyzed the complete mitochondrial genome sequence of *L. ikeae* and compared, analyzed the protein-coding gene (PCG) sequences in the mitochondrial genomes of 28 Sicydiinae species, and constructed a phylogenetic tree based on them. We systematically discuss the phylogenetic relationships among Sicydiinae, laying a foundation for future research on the phylogenetic relationships in this subfamily.

## 2. Materials and Methods

### 2.1. Mitochondrial Genome Sequencing and Assembly

*Lentipes ikeae* was collected in July 2021 at the New World of Flowers, Birds, Fish, and Insects in Fangcun, Liwan District, Guangzhou City, Guangdong Province, China (23°3′47.69″ N, 113°12′19.42″ E). The fish specimens were soaked in anhydrous ethanol and stored at −20 °C in the fish specimen library of the Department of Ecology, Jinan University, Guangzhou City, Guangdong Province, China. Total genomic DNA was extracted from back muscle tissues using the improved CTAB extraction protocol [11] and sent to Biozero Biotechnology Co., Ltd. (Shanghai, China) for 350-bp small-fragment library construction and high-throughput sequencing. PE150 sequencing (paired-end 150 bp: we performed separate sequencing of DNA samples to generate two reads with a length of 150 bp) was conducted on the Illumina HiSeq X Ten sequencing platform and the sequencing data subjected to quality control; effective read segments larger than 10 GB were retained. The read segments from each sample were assembled de novo using SPAdes v3.0.0 to obtain the complete mitochondrial genome sequence [12,13].

### 2.2. Mitochondrial Genome Sequence Annotation and Comparative Genome Analysis

FASTA files of the *L. ikeae* mitochondrial genome sequence were imported into MITOS (http://mitos2.bioinf.uni-leipzig.de/index.py, accessed on 1 January 2022) for preliminary annotation [14]. PCG and ribosomal (r)RNA gene sequences were compared with those of related species and corrected using Genesis v4.8.5 [15]. tRNAscan-SE v2.0 was used to predict transfer (t)RNA genes and their secondary structures [16]. The *L. ikeae* mitochondrial genome’s AT content, AT skew, GC skew, and relative synonymous codon usage (RSCU) were calculated in PhyloSuite v1.2.1 [17]. Base composition skewness was calculated as follows: AT skew = (A − T)/(A + T), GC skew = (G − C)/(G + C).

### 2.3. Phylogenetic Analysis

To fully explore the evolutionary position of *L. ikeae* in Sicydiinae, phylogenetic analysis was conducted using mitochondrial sequence data from 28 representative Sicydiinae species reported in GenBank and the complete mitochondrial whole genome of *L. ikeae*. *Acanthogobius flavimanus* (GenBank accession MW271007; Gobionellinae) was selected as the outgroup; phylogenetic trees were constructed on the basis of a 13 PCG sequence dataset. For the PCGs, we used MAFFT for multiple sequence alignment [18] and Gblocks [19] to remove vacancies and fuzzy alignment sites. The multiple sequence alignment results of individual genes were combined using SequenceMatrix [20] to obtain a dataset of the 13 PCGs. Dataset saturation was evaluated using DAMBE v5.0 [21], which revealed that all datasets were non-saturated (Iss < lss.cSym or lss.cAsym, *p* < 0.05). We used PartitionFinder2 [22] to select the evolutionary model corresponding to the optimal dataset partition. Phylogenetic analysis was conducted on the PhyloSuite platform. A maximum likelihood (ML) phylogenetic tree was constructed using IQ-TREEv1.6.8 [23], with 50,000 bootstraps to evaluate the branching node reliability, and the bootstrap value of each branch was calculated. We used MrBayes v3.2.6 [24] to construct a Bayesian inference (BI) phylogenetic tree. Four independent Markov chains were set to run simultaneously for 200 million generations, with sampling once every 1000 generations. When the effective sampling size was ≥200 and the average standard deviation of the split frequency was ≤0.01, assuming that all runs had reached saturation and that the MrBayes results had converged, 25% of the aging samples were discarded, the remaining samples were used to construct a unified tree, and the Bayesian posterior probability values for each node were calculated. We used iTOL (https://itol.embl.de/, accessed on 1 January 2022) [25] for the beautification of the final phylogenetic tree.

## 3. Results

### 3.1. Mitochondrial Genome Structure

**Figure 1:**
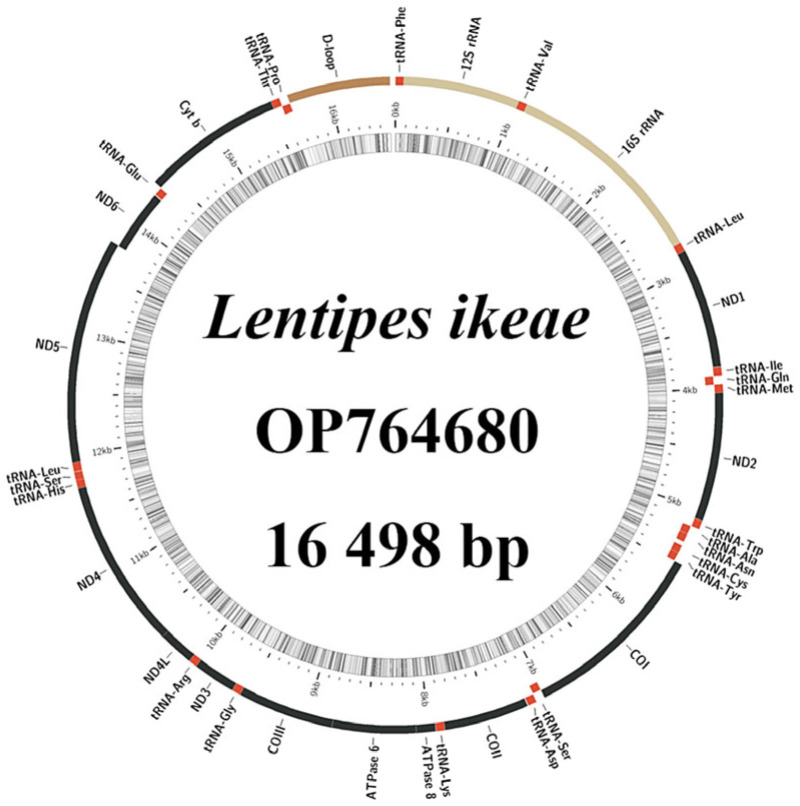
*Lentipes ikeae* mitochondrial genome structure.

The complete mitochondrial genome of *L. ikeae* encodes 37 genes (13 PCGs, 22 tRNA genes, and two rRNA genes) and a circular DNA molecule composed of a control region, with a total length of 16,498 bp (Figure 1). The difference in the mitochondrial genome length between *L. ikeae* and the other three species (16,496–16,499 bp) in this genus is very small. One PCG and eight tRNA genes are located on the light (L-)strand, and 28 genes on the heavy (H-)strand. Gene rearrangement is not observed. The *L. ikeae* mitochondrial genome genes are tightly arranged, and there are six gene overlaps and 11 gene intervals with lengths of 1–7 bp. There is a 34-bp gap between the tRNA-Asn and tRNA-Cys genes (Table 1). Among the 13 PCGs, only *COI* has the start codon GTG, whereas the other genes have the ATG start codon. Except for *ATP6*, *COII*, *COIII*, *Cytb*, *ND3*, and *ND4*, which have an incomplete termination codon T or TA, the other PCGs have a TAA/TAG termination codon. The 16S rRNA and 12S rRNA genes are 1682 and 850 bp, respectively, in size. The non-coding D-loop region is located between the tRNA-Pro and tRNA-Phe genes and has a length of 843 bp.

### 3.2. Mitochondrial Genome Base Preference and Relative Synonymous Codon Usage Frequency

**Figure 2:**
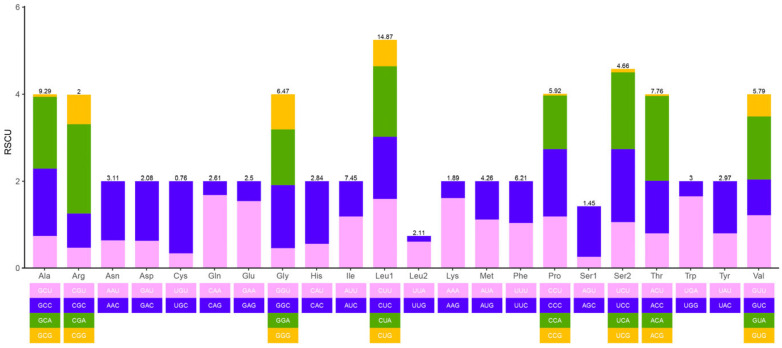
RSCU in PCGs in the mitochondrial genome of *Lentipes ikeae.* Different colors correspond to different third codons.

The complete mitochondrial genome of *L. ikeae* has a clear AT preference, with total AT content of 54.9%. The mitochondrial genomes of the other three species in this genus also have a clear AT preference (54.9–55.0%). The AT content of the PCGs and tRNA and rRNA genes is 54.3%, 55.4%, and 54.9%, respectively, indicating that different positions in the mitochondrial genome of *L. ikeae* have different base usage preferences. The value of the AT skew of the mitochondrial genome is 0.035 and that of GC skew is −0.264, indicating that the mitochondrial genome of *L. ikeae* preferentially uses A and C bases. We found significant differences in the AT content of different coding sites in the PCGs, with the AT content being significantly higher in the second and third sites than in the first site (Table 2). The high AT content was also reflected in the use of relatively synonymous codons in the PCGs. Codons rich in G and C bases, such as CCG, ACG, GCG, and UCG, had RSCU values below 1 and a relatively low usage frequency, whereas codons rich in A and T bases, such as ACA and CAA, were the most commonly used (Figure 2). The RSCU values of different codons were found to greatly vary, with a preference for the use of codons rich in A and T bases and codons ending with A and T bases in the PCGs of the *L. ikeae* mitochondrial genome.

### 3.3. Phylogenetic Relationships

**Figure 3:**
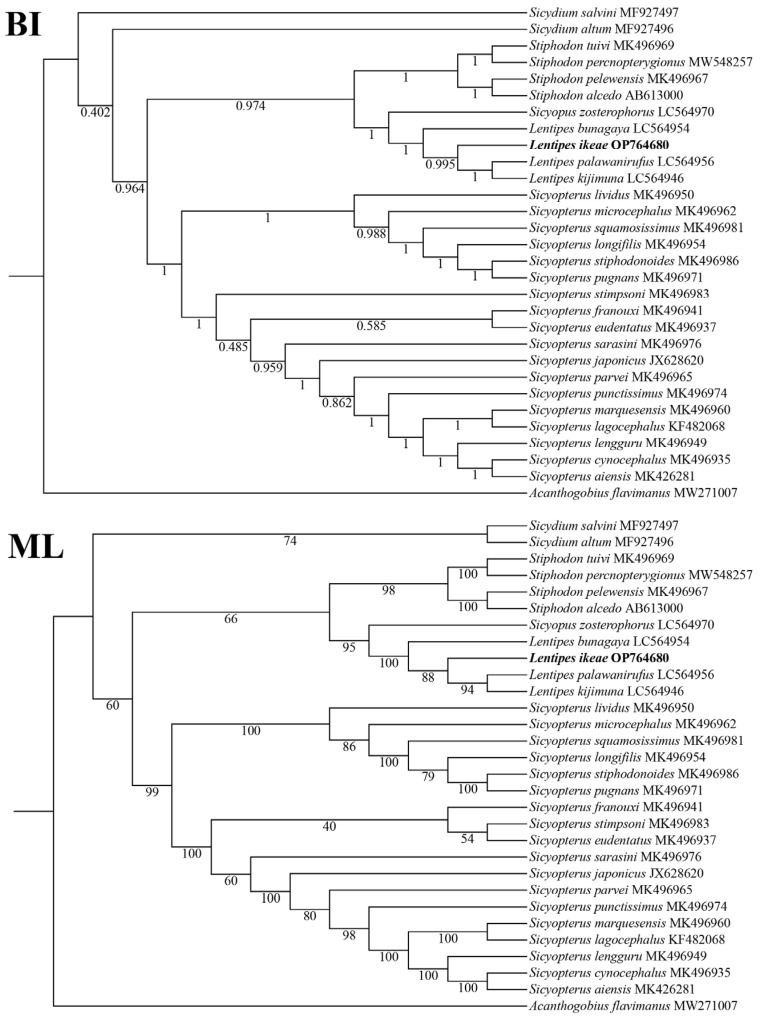
Phylogenetics relationships among Sicydiinae species based on nucleotide sequences of 13 mitochondrial PCGs.

The topological structures of the ML and BI trees based on the 13 PCG dataset were basically the same (Figure 3), with both trees supporting that *Sicydium*, *Sicyopterus*, *Lentipes*, and *Stiphodon* are monophyletic groups, and *Lentipes*, *Sicyopus*, and *Stiphodon* are sister groups. *Sicyopus* and *Lentipes* first form a sister group and then form a branch with Stiphodon. *L. ikeae* and (*Lentipes palawanirufus* + *Lentipes kijimuna*) first form a branch and then cluster with *Lentipes bunagaya*, supporting the taxonomic status of *L. ikeae*. The phylogenetic relationships among Sicydiinae are as follows: *Sicydium* + (*Stiphodon* + (*Sicyopus* + *Lentipes*)) + *Sicyopterus*.

## 4. Discussion

The mitochondrial genome of *L. ikeae* has a total length of 16,498 bp and encodes 22 tRNA genes, 13 PCGs, two rRNAs, and one D-loop. Other *Lentipes* mitochondrial genomes have similar structures [4]. The A+T base content (54.9%) is greater than that of G+C bases (45.1%), revealing an AT preference, as observed in other fish, such as *Oliotius* Kottelat [26] and *Rhinogobius* [27].

In the mitochondrial genome of *L. ikeae*, PCGs are present on both the H- and L-strands. Brown [28] reported that the PCGs on the H-strand are incompletely protected because the H-strand is often in a hydrolyzed, single-stranded state. Like in *Oliotius* Kottelat [26], the majority of PCGs in the *L. ikeae* mitochondrial genome are located on the H-strand, rendering them prone to hydrolysis and oxidation. The *ND6* gene located on the L-strand is substantially more stable, reflecting the diversity and importance of *ND6* genes.

*L. ikeae* has 22 tRNA genes, ranging in length from 67 bp to 76 bp and totaling 15,57 bp, similar to *Rhinogobius* [27] and *Lentipes* [4]. The mitochondrial genome of *L. ikeae* contains two rRNAs, 16S rRNA and 12S rRNA, which are not isolated or overlapping with adjacent genes. Repetitive sequences, partially inserted sequences, and sequences contained in the mitochondrial genome are all characteristics of species evolution [5].

In the phylogenetic trees, *L. ikeae* clustered together with three other species in the genus, *Lentipes palawanirufus*, *Lentipes kijimuna*, and *Lentipes bunagaya*, and showed the closest genetic relationship with *Sicyopus zosterophorus*. The monophyletic nature of the four genera *Sicydium*, *Sicyopterus*, *Lentipes*, and *Stiphodon* was well supported. Related genes in mitochondrial genomes are currently widely used in phylogenetic research and species classification. Kim et al. [29] constructed a phylogenetic tree based on mitochondrial 12S rRNA genes to study the evolutionary status of *Acanthogobius hasta*. With the widespread use of molecular biology methods, domestic and foreign researchers are increasingly studying the phylogenetics of Gobiidae fish. Agorreta et al. [30] analyzed the phylogenetic relationships of 222 European Gobioidei fish species. Thacker et al. [31] confirmed that Trachinoidei is the sister lineage of Gobioidei and studied the systematic distribution of Gobioidei in Acanthomorpha [32]. In general, the evolutionary speed of mitochondrial genes is greater than that of species, and different geographical environments and lifestyle habits can lead to DNA variations in species. To fully unravel the evolution of the *L. ikeae* mitochondrial genome, further research on the mitochondrial genes is needed.

## Figures and Tables

**Table 1:** Organization of the *Lentipes ikeae* mitochondrial genome.

Gene	Size	Position	Intergenic Nucleotides	Start Codon	Stop Codon	Strand
tRNA-Phe	68	1–68	0			H
12S rRNA	950	69–1018	0			H
tRNA-Val	72	1019–1090	0			H
16S rRNA	1682	1091–2772	0			H
tRNA-Leu2	76	2773–2848	0			H
ND1	975	2849–3823	0	ATG	TAA	H
tRNA-Ile	70	3827–3896	3			H
tRNA-Gln	71	3896–3966	–1			L
tRNA-Met	69	3966–4034	–1			H
ND2	1047	4035–5081	0	ATG	TAA	H
tRNA-Trp	71	5083–5153	1			H
tRNA-Ala	69	5156–5224	2			L
tRNA-Asn	73	5226–5298	1			L
tRNA-Cys	67	5333–5399	34			L
tRNA-Tyr	71	5400–5470	0			L
COI	1554	5472–7025	1	GTG	TAA	H
tRNA–Ser2	71	7026–7096	0			L
tRNA-Asp	72	7100–7171	3			H
COII	691	7177–7867	5	ATG	T	H
tRNA-Lys	75	7868–7942	0			H
ATP8	165	7944–8108	1	ATG	TAA	H
ATP6	683	8102–8784	–7	ATG	TA	H
COIII	784	8785–9568	0	ATG	T	H
tRNA-Gly	72	9569–9640	0			H
ND3	349	9641–9989	0	ATG	T	H
tRNA-Arg	69	9990–10,058	0			H
ND4L	297	10,059–10,355	0	ATG	TAA	H
ND4	1381	10,349–11,729	–7	ATG	T	H
tRNA-His	69	11,730–11,798	0			H
tRNA-Ser1	68	11,799–11,866	0			H
tRNA-Leu1	73	11,870–11,942	3			H
ND5	1839	11,943–13,781	0	ATG	TAA	H
ND6	522	13,778–14,299	–4	ATG	TAG	L
tRNA-Glu	69	14,300–14,368	0			L
Cytb	1141	14,374–15,514	5	ATG	T	H
tRNA-Thr	72	15,515–15,586	0			H
tRNA-Pro	70	15,586–15,655	–1			L
D-loop	843	15,656–16,498	0			H

**Table 2:** Nucleotide composition of the *Lentipes ikeae* mitochondrial genome.

Region	Size (bp)	T	C	A	G	AT (%)	GC (%)	AT Skew	GC Skew
Full genome	16,498	26.5	28.5	28.4	16.6	54.9	45.1	0.035	−0.264
PCGs	11,421	28.5	29.6	25.8	16.1	54.3	45.7	−0.050	−0.295
1st codon position	3807	19.9	28.2	25.9	26.1	45.7	54.3	0.132	−0.039
2nd codon position	3807	40.6	27.6	18.2	13.7	58.8	41.2	−0.382	−0.338
3rd codon position	3807	25.0	33.0	33.4	8.6	58.3	41.7	0.144	−0.586
tRNAs	1557	27.2	20.8	28.2	23.8	55.4	44.6	0.019	0.068
rRNAs	2632	21.3	24.4	33.6	20.7	54.9	45.1	0.224	−0.082
D-Loop	843	31.2	22.1	31.4	15.3	62.6	37.4	0.004	−0.181

## Data Availability

The complete mitochondrial genome sequence and annotations of *Lentipes ikeae* are available in GenBank with accession number OP764680.
